# CCL21/CCR7 Promotes G_2_/M Phase Progression via the ERK Pathway in Human Non-Small Cell Lung Cancer Cells

**DOI:** 10.1371/journal.pone.0021119

**Published:** 2011-06-16

**Authors:** Ying Xu, Lifeng Liu, Xueshan Qiu, Lili Jiang, Bo Huang, Haiying Li, Zixuan Li, Wenting Luo, Enhua Wang

**Affiliations:** 1 Department of Pathology, First Affiliated Hospital and College of Basic Medical Sciences, China Medical University, Shenyang, Liaoning, China; 2 Institute of Pathology and Pathophysiology, China Medical University, Shenyang, Liaoning, China; 3 Department of Orthopaedics, First Affiliated Hospital of China Medical University, Shenyang, Liaoning, China; University of Pennsylvania, United States of America

## Abstract

C-C chemokine receptor 7 (CCR7) contributes to the survival of certain cancer cell lines, but its role in the proliferation of human non-small cell lung cancer (NSCLC) cells remains vague. Proliferation assays performed on A549 and H460 NSCLC cells using Cell Counting Kit-8 indicated that activation of CCR7 by its specific ligand, exogenous chemokine ligand 21 (CCL21), was associated with a significant linear increase in cell proliferation with duration of exposure to CCL21. The CCL21/CCR7 interaction significantly increased the fraction of cells in the G_2_/M phase of the cell cycle as measured by flow cytometry. In contrast, CCL21/CCR7 had no significant influence on the G_0_/G_1_ and S phases. Western blot and real-time PCR indicated that CCL21/CCR7 significantly upregulated expression of cyclin A, cyclin B1, and cyclin-dependent kinase 1 (CDK1), which are related to the G_2_/M phase transition. The expression of cyclin D1 and cyclin E, which are related to the G_0_/G_1_ and G_1_/S transitions, was not altered. The CCL21/CCR7 interaction significantly enhanced phosphorylation of extracellular signal-regulated kinase (P-ERK) but not Akt, as measured by Western blot. LY294002, a selective inhibitor of PI3K that prevents activation of the downstream Akt, did not weaken the effect of CCL21/CCR7 on P-ERK. Coimmunoprecipitation further confirmed that there was an interaction between P-ERK and cyclin A, cyclin B1, or CDK1, particularly in the presence of CCL21. CCR7 small interfering RNA or PD98059, a selective inhibitor of MEK that disrupts the activation of downstream ERK, significantly abolished the effects of exogenous CCL21. These results suggest that CCL21/CCR7 contributes to the time-dependent proliferation of human NSCLC cells by upregulating cyclin A, cyclin B1, and CDK1 potentially via the ERK pathway.

## Introduction

C-C chemokine receptor 7 (CCR7) is expressed on all naive T-cells and on some memory T-cells, B-cells, and mature dendritic cells [1]. Upon interaction with its ligands, chemokine ligand 19 (CCL19) or chemokine ligand 21 (CCL21) [2], CCR7 contributes to lymphocyte trafficking and homing to lymph nodes during immune and inflammatory reactions [3]–[5]. CCR7 is highly expressed in non-small cell lung cancer (NSCLC), breast cancer, and squamous cell carcinoma of the head and neck and is responsible for mediating metastasis in certain cancer cells lines [6]–[15]. To date, the role of CCR7 in the proliferation of human NSCLC cells has not been elucidated.

Activation of CCR7 can increase phosphorylation of extracellular signal-regulated kinase (P-ERK) or Akt (P-Akt) via Gi proteins to enhance cell proliferation or survival [16]–[20]. ERK belongs to the mitogen-activated protein kinase (MAPK) family, which also includes c-Jun N-terminal kinase (JNK) and p38. The ERK cascade, activated by mitogenic stimuli, is critical for proliferation and survival [21], [22] and is required for normal progression into mitosis [23], [24]. The JNK and p38 pathways are activated in response to chemicals and environmental stress [25]–[27]. Akt (also known as Akt1), a mediator of growth factor-induced cell survival [28]–[30], may promote cell proliferation via phosphorylation [31].

The purpose of this study was to examine the effect and regulatory mechanism of the CCL21/CCR7 interaction on the proliferation of A549 and NCI-H460 (H460) human NSCLC cells. Here, we demonstrated that CCL21/CCR7 contributed to the time-dependent proliferation of human NSCLC cells by upregulating the expression of cyclin A, cyclin B1, and CDK1 via the ERK pathway. Information garnered from this study lends insight to the mechanisms of survival of CCR7-mediated cancer cells and has implications for treatment targets in NSCLC.

## Results

**Figure 1:**
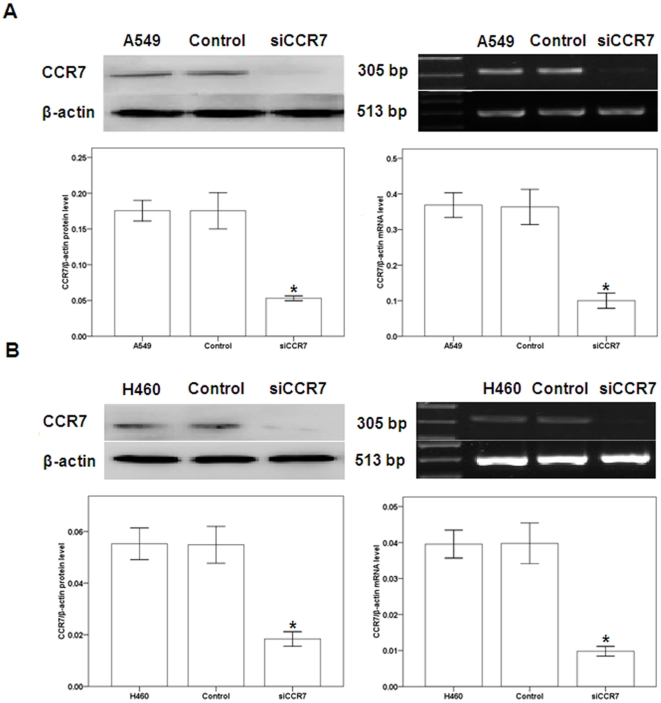
Efficiency of CCR7 siRNA in A549 or H460 cells. A549 (A) and H460 (B) cells were transfected with control siRNA or CCR7 siRNA (siCCR7). After transfection, the expression of CCR7 protein (a) and mRNA (b) was evaluated using Western blot (a) and RT-PCR (b) and compared to untransfected A549 or H460 cells. Each bar represents the mean ± SD of three independent experiments. *p<0.05, compared with control cells.

CCL21/CCR7 promotes proliferation of A549 and H460 cells. In a previous study, we identified a higher CCR7 expression level in A549 and H460 human NSCLC cell lines compared with other cell lines [32]. To investigate the role of CCR7 in the functioning of A549 and H460 cells, CCR7 activation and inhibition were induced with exogenous CCL21 and with CCR7 small interfering RNA (siRNA), respectively. After transfection with CCR7 siRNA (siCCR7) or control siRNA, the expression of CCR7 was evaluated using Western blot and reverse transcriptase (RT)-PCR. We found that siCCR7 significantly downregulated the protein and mRNA levels of CCR7, compared with control siRNA (Figure 1).

**Figure 2:**
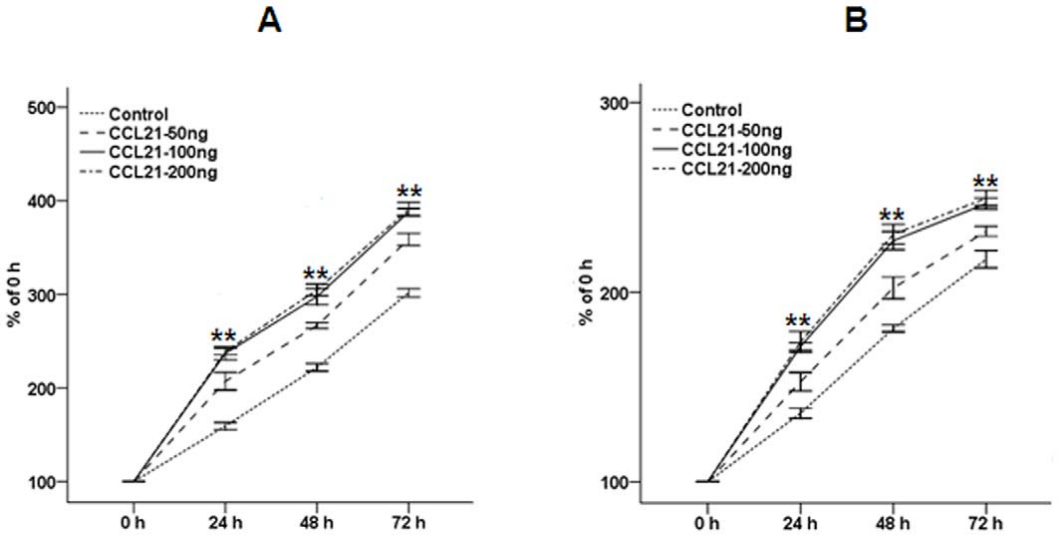
Effect of CCL21 at various concentrations on proliferation of A549 and H460 cells. A549 (A) and H460 (B) cells were treated with CCL21 (50, 100 or 200 ng/mL) for 24, 48, or 72 h, and cell vitality was estimated using the CCK-8 assay. Each bar represents the mean ± SD of three independent experiments. **p<0.01, compared with cells treated with CCL21 (50 ng/mL).

**Figure 3:**
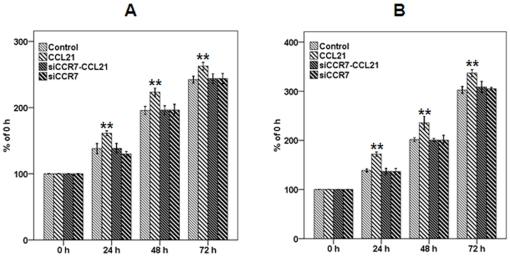
Effect of CCL21/CCR7 on proliferation of A549 and H460 cells. A549 (A) and H460 (B) cells were treated with CCL21 (100 ng/mL) for 24, 48, or 72 h, and cell vitality was estimated using the CCK-8 assay. Each bar represents the mean ± SD of three independent experiments. **p<0.01, compared with control cells.

To determine the effect of CCL21/CCR7 on cell proliferation, the CCK-8 assay was performed on A549 and H460 cells. According to the published data [33], [34] and the results of our preliminary experiment, at 100 ng/mL concentrations CCL21 significantly promoted cell proliferation, compared with 50 ng/mL concentrations, while there were no significant difference between 100 ng/mL and 200 ng/mL concentrations (Figure 2). Therefore, at 100 ng/mL concentrations CCL21 was used in following experiment. The CCL21/CCR7 interaction significantly promoted cell proliferation, whereas siCCR7 significantly abrogated the action of CCL21 (Figure 3). siCCR7 alone had no significant effect on cell proliferation, compared with control cells. Significant differences were observed between all time point examined (all p<0.01), indicating a linear increase in proliferation with increasing exposure times to CCL21 (all p<0.01).

CCL21/CCR7 augments the proportion of cells in G_2_/M. To verify whether the action of CCL21/CCR7 on the proliferation of A549 and H460 cells is associated with a change in cell cycle distribution, cell cycle analysis was performed using flow cytometry. The CCL21/CCR7 interaction significantly enhanced the proportion of cells in the G_2_/M phase, whereas there was no significant effect of this interaction on the proportion of cells in G_0_/G_1_ or the S phase, compared with control cells (Table 1). siCCR7 significantly abolished this effect of CCL21, whereas siCCR7 alone had no significant effect on cell cycle distribution.

**Table 1:** Effect of CCL21/CCR7 on cell cycle distribution in A549 and H460 cells.

Group	G_0_/G_1_ phase	S phase	G_2_/M phase
	Mean ± SD (%)	Mean ± SD (%)	Mean ± SD (%)
(A549) Control	74.35±9.67	23.42±12.29	2.23±2.82
(A549) CCL21	66.92±1.23	21.60±2.91	11.48±2.64*
(A549) siCCR7-CCL21	69.08±10.08	23.90±9.28	6.60±2.46
(A549) siCCR7	75.73±2.32	18.09±3.67	6.19±2.45
(H460) Control	61.50±2.70	31.33±3.41	7.17±1.63
(H460) CCL21	60.38±6.85	27.08±4.13	12.54±2.75*
(H460) siCCR7-CCL21	69.25±4.99	21.40±3.23	6.02±2.53
(H460) siCCR7	67.57±2.40	28.00±4.10	4.63±1.20

A549 and H460 cells were treated with CCL21 (100 ng/mL) for 24 h, and cell cycle distribution was estimated using flow cytometry. Data were present as mean ± SD of three independent experiments.

*p<0.05, compared with control cells.

**Figure 4:**
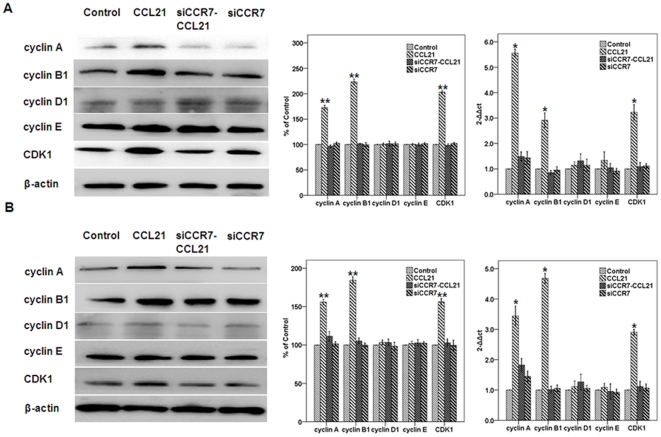
Effect of CCL21/CCR7 on the expression of cyclins and cyclin-dependent kinases in A549 and H460 cells. A549 (A) and H460 (B) cells were treated with CCL21 (100 ng/mL) for 24 h, and the protein (a) and mRNA (b) levels of cyclins A, B1, D1, and E and of CDK1 were estimated using Western blot (a) and real-time PCR (b). Each bar represents the mean ± SD of three independent experiments. *p<0.05 or **p<0.01, compared with control cells.

CCL21/CCR7 upregulates the expression of cyclin A, cyclin B1, and CDK1. To determine the possible mechanism by which CCL21/CCR7 influences the G_2_/M phase distribution in A549 and H460 cells, the expression of cyclins and cyclin-dependent kinase 1 (CDK1) was assessed using Western blot and real-time PCR. Compared with control cells, the CCL21/CCR7 interaction significantly upregulated the protein and mRNA levels of cyclin A, cyclin B1, and CDK1, which are related to G_2_/M phase progression (Figure 4). siCCR7 significantly abrogated the effects of CCL21, whereas siCCR7 alone had no significant effect on cyclin or CDK1 expression. CCL21 had no significant effect on the levels of cyclin D1 or cyclin E, which are related to G_0_/G_1_ and the G_1_/S transition.

**Figure 5:**
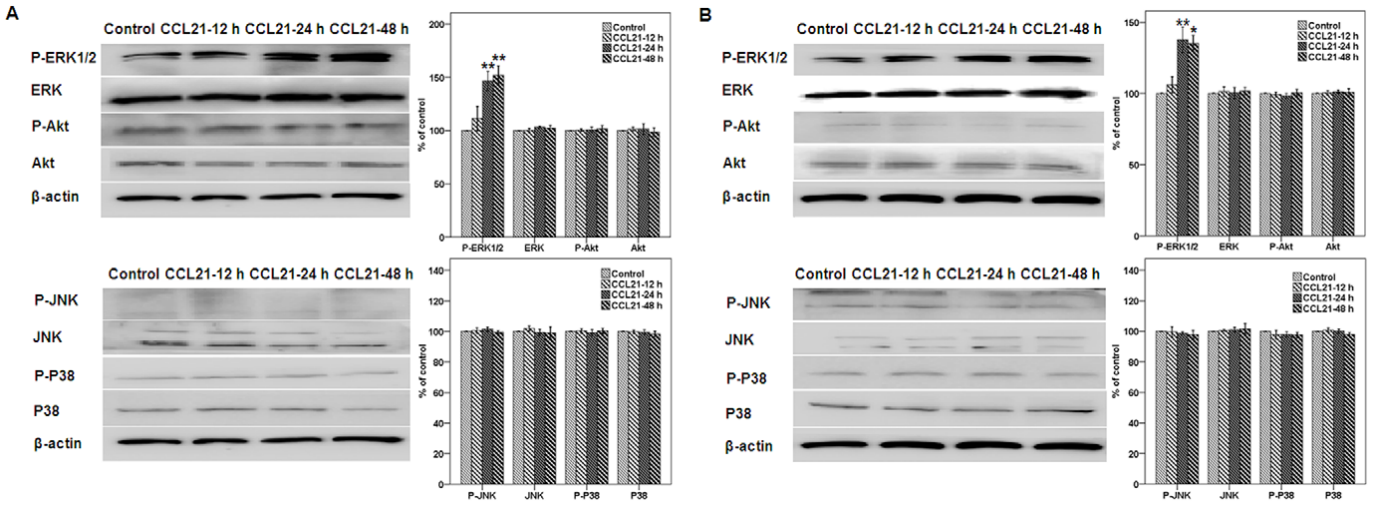
Effect of CCL21/CCR7 on the expression of ERK, JNK, p38, and Akt in A549 and H460 cells. A549 (A) and H460 (B) cells were treated with CCL21 (100 ng/mL) for 12, 24, or 48 h, and normal and phosphorylated (P-) expression levels were estimated by Western blot. Each bar represents the mean ± SD of three independent experiments. *p<0.05 or **p<0.01, compared with control cells.

**Figure 6:**
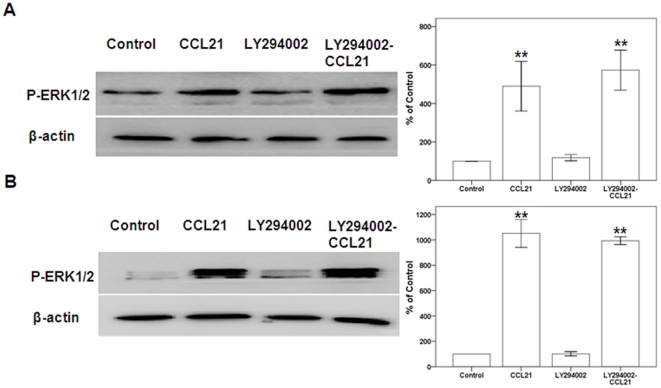
Effects of CCL21/CCR7 on the expression of P-ERK after inhibiting activation of Akt in A549 and H460 cells. A549 (A) and H460 (B) cells were treated with CCL21 (100 ng/mL) for 24 h after LY294002, a selective inhibitor of PI3K that consequently prevents activation of the downstream Akt, was applied for 1 h. The expression of P-ERK was estimated using Western blot. Each bar represents the mean ± SD of three independent experiments. **p<0.01, compared with control cells.

CCL21/CCR7 upregulates the expression of P-ERK but not P-Akt. Others have reported that CCR7 may increase the phosphorylation of ERK, JNK, or Akt, which are related to cell survival [16]–[20], [33], [35]–[42]. To verify whether the CCL21/CCR7 interaction may also enhance the expression of MAPK family members and Akt in A549 and H460 cells, the expression of these components was assessed using Western blot. The CCL21/CCR7 interaction significantly upregulated the expression of P-ERK at 24 h and 48 h, whereas there was no significant impact on the expression of ERK (Figure 5). CCL21/CCR7 had no significant influence on the expression or phosphorylation of JNK, p38, or Akt. Because Akt is possibly upstream of ERK [43], A549 and H460 cells were treated with CCL21 for 24 h after a 1-h exposure to LY294002, a selective inhibitor of PI3K that inhibits activation of the downstream Akt pathway. Following this treatment, CCL21/CCR7 still significantly upregulated the expression of P-ERK (Figure 6).

**Figure 7:**
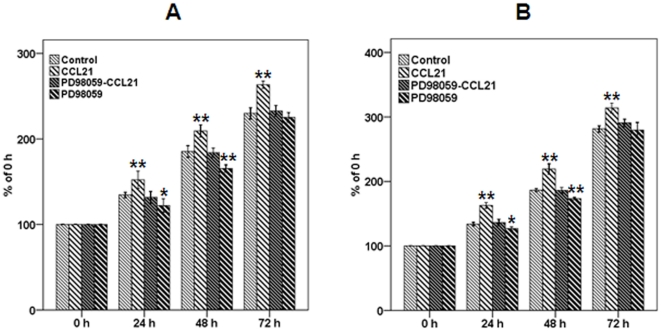
Effect of CCL21/CCR7 on proliferation of A549 and H460 cells after inhibiting ERK activation. A549 (A) and H460 (B) cells were treated with CCL21 (100 ng/mL) for 24, 48, or 72 h after PD98059, a selective inhibitor of MEK that disrupts activation of downstream ERK, was applied for 1 h. Cell vitality was estimated using the CCK-8 assay. Each bar represents the mean ± SD of three independent experiments. *p<0.05 or **p<0.01, compared with control cells.

**Figure 8:**
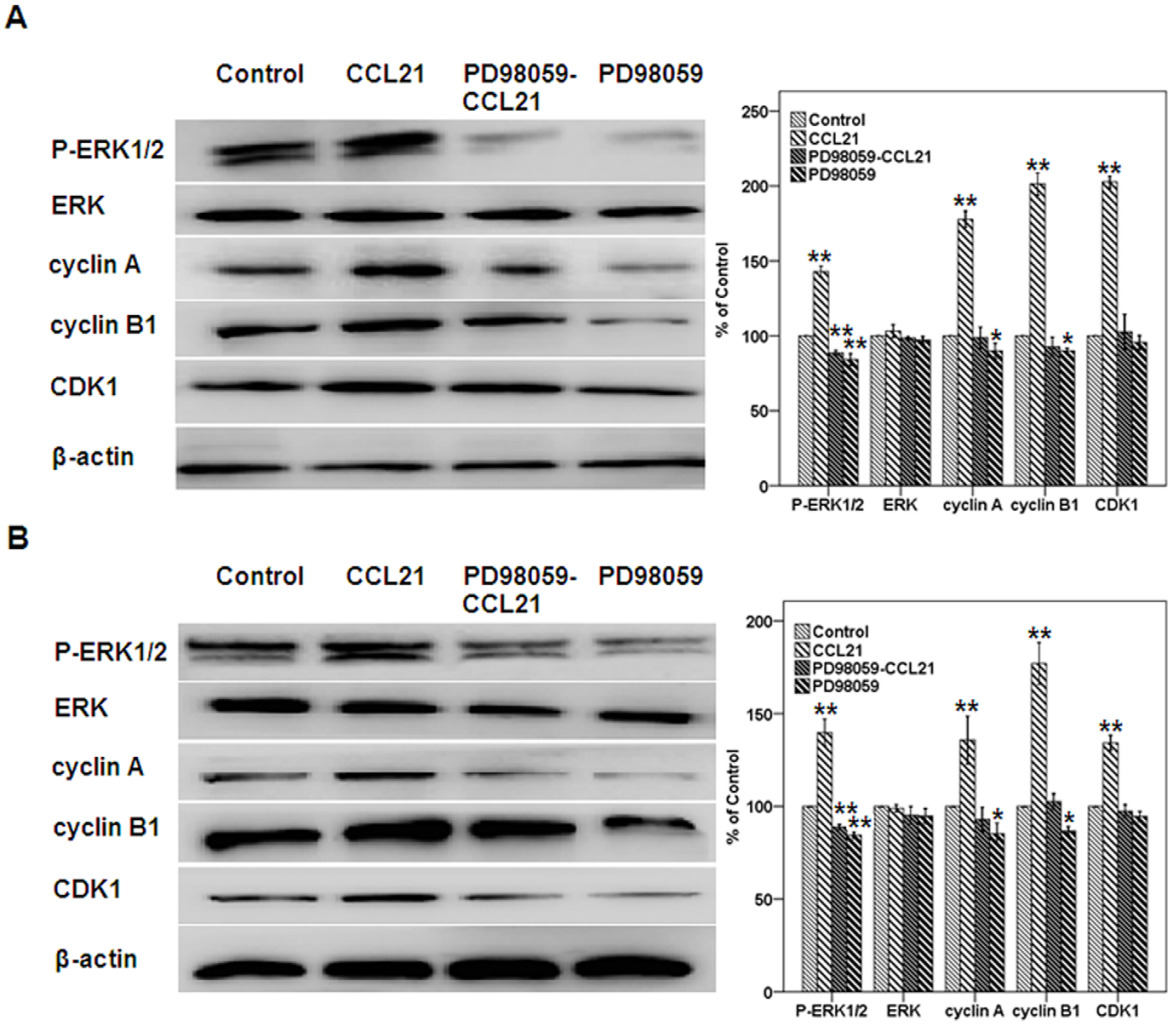
Effect of CCL21/CCR7 on the expression of P-ERK, cyclin A, cyclin B1, and CDK1 after inhibiting the activation of ERK. A549 (A) and H460 (B) cells were treated with CCL21 (100 ng/mL) for 24 h after PD98059, a selective inhibitor of MEK that disrupts activation of downstream ERK, was applied for 1 h. The expression levels of these components were estimated using Western blot. Each bar represents the mean ± SD of three independent experiments. *p<0.05 or **p<0.01, compared with control cells.

Inhibition of P-ERK abolishes the impellent effects of CCL21/CCR7 on proliferation and G_2_/M phase progression. To verify whether PD98059, a selective inhibitor of MEK that disrupts activation of downstream ERK, can abolish the effects of CCL21/CCR7 on the proliferation and G_2_/M phase progression of A549 and H460 cells, cell viability and cell cycle distribution assays were performed using CCK-8 and flow cytometry, respectively. PD98059 significantly abrogated the effects of CCL21/CCR7 on cell proliferation and the G_2_/M phase progression (Figure 7 and Table 2, respectively). PD98059 also abolished the influence of CCL21/CCR7 on the expression of P-ERK, cyclin A, cyclin B1, and CDK1 (Figure 8). In addition, PD98059 alone had a significant inhibitory effect on the cell proliferation, the G_2_/M phase progression and the expression of P-ERK, cyclin A, and cyclin B1 (Figure 7, Table 2, and Figure 8, respectively).

**Table 2:** Effect of CCL21/CCR7 on cell cycle distribution after inhibiting ERK in A549 and H460 cells.

Group	G_0_/G_1_ phase	S phase	G_2_/M phase
	Mean ± SD (%)	Mean ± SD (%)	Mean ± SD (%)
(A549) Control	80.49±0.67	14.57±1.48	4.94±0.91
(A549) CCL21	68.58±3.17	20.21±0.40	11.48±2.64**
(A549) PD98059-CCL21	73.06±6.94	22.11±7.42	4.84±0.58
(A549) PD98059	76.65±11.28	24.64±5.83	2.05±0.31*
(H460) Control	65.73±1.72	27.95±0.94	6.33±1.76
(H460) CCL21	58.58±8.68	29.41±6.16	12.01±2.70**
(H460) PD98059-CCL21	67.80±3.00	26.91±3.17	5.29±1.29
(H460) PD98059	67.07±2.17	30.50±3.02	2.43±1.18*

A549 and H460 cells were treated with CCL21 (100 ng/mL) for 24 h after PD98059, a selective inhibitor of MEK that disrupts activation of downstream ERK, was used for 1 h. After treatment, cell cycle distribution was estimated using flow cytometry. Data were present as mean ± SD of three independent experiments.

*p<0.05 or

**p<0.01, compared with control cells.

**Figure 9:**
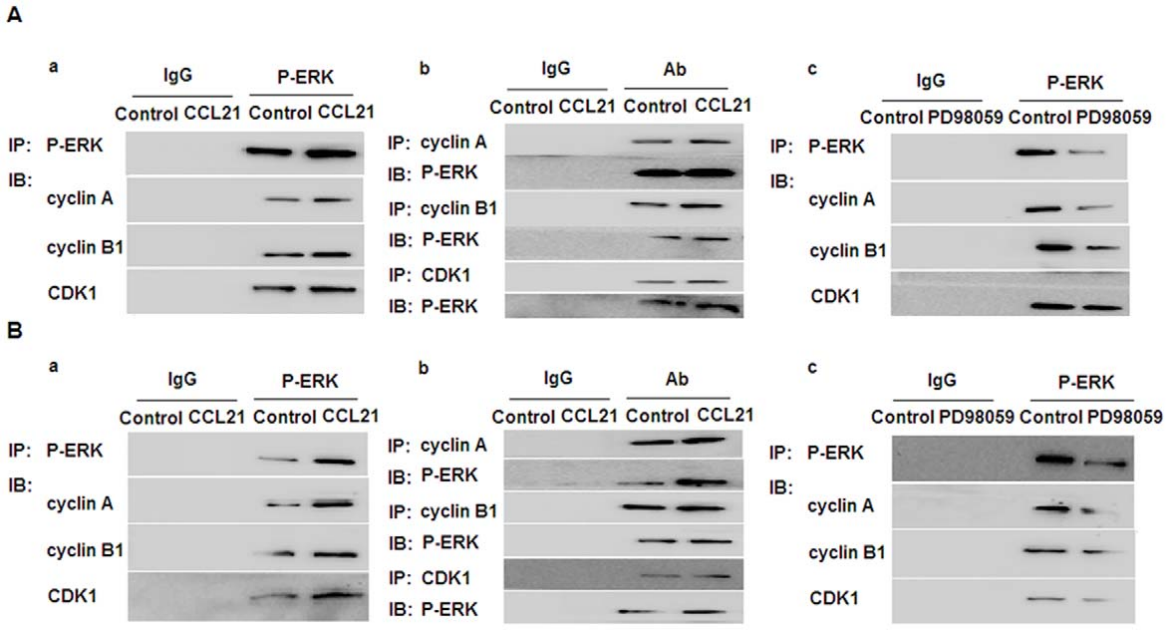
The interaction between P-ERK and cyclin A, cyclin B1, or CDK1 in the absence or presence of CCL21 or PD98059. A549 (A) and H460 (B) cells in the absence or presence of CCL21 (100 ng/mL) for 24 h were subjected to immunoprecipitation with antibodies against P-ERK or IgG, followed by Western blotting for cyclin A, cyclin B1, and CDK1 (a). Reciprocal immunoprecipitation with antibodies against cyclin A, cyclin B1, CDK1, or IgG were analyzed by Western blotting for P-ERK (b). A549 and H460 cells in the absence or presence of PD98059 for 1 h were subjected to immunoprecipitation with P-ERK or IgG antibodies followed by Western blotting for cyclin A, cyclin B1, and CDK1 (c).

P-ERK, induced by CCL21/CCR7, interacts with cyclin A, cyclin B1, and CDK1. To further identify whether there is an interaction between P-ERK and cyclin A, cyclin B1, or CDK1, coimmunoprecipitation was performed. A549 and H460 cells in the absence or presence of CCL21 for 24 h were subjected to immunoprecipitation with antibodies against P-ERK or IgG, followed by Western blotting for cyclin A, cyclin B1, and CDK1. A pronounced, specific interaction between P-ERK and cyclin A, cyclin B1, or CDK1 was observed, especially when the cells were treated with CCL21 for 24 h (Figure 9a). Reciprocal immunoprecipitation with antibodies against cyclin A, cyclin B1, CDK1, or IgG was assessed by Western blotting for P-ERK, and again, the interaction between P-ERK and cyclin A, cyclin B1, or CDK1 was salient, especially in the presence of CCL21 (Figure 9b). A549 and H460 cells in the absence or presence of PD98059 for 1 h were subjected to immunoprecipitation with antibodies against P-ERK or IgG, followed by Western blotting for cyclin A, cyclin B1, and CDK1. The interaction between P-ERK and cyclin A, cyclin B1, or CDK1 was weakened in response to PD98059 exposure (Figure 9c).

## Discussion

Several studies have documented that the activation of CCR7 is responsible for mediating survival of certain cancer cell lines by promoting migration and proliferation or by inhibiting apoptosis [33], [35]–[42]. However, the role of CCR7 in the proliferation of human NSCLC cells has not been well documented. In the present study, we confirmed that the CCL21/CCR7 interaction can significantly enhance human NSCLC cell proliferation in a time-dependent manner, involving the upregulation of cyclin A, cyclin B1, and CDK1, possibly via the ERK, but not the Akt, pathway.

Consistent with previous studies using different cell models [16], [37], [40], the activation of CCR7 with CCL21 promoted cell proliferation. In contrast to our current findings, a prior study suggested that murine CCL21 had no significant action on the proliferation of A549 cells [44]. A possible explanation for the discrepancy would be that mouse CCL21 can interact with CXCR3 but not CCR7, whereas human CCL21 can bind CCR7 but not CXCR3 [45]. This suggests that activation of CXCR3 does not affect proliferation of A549 cells.

Traditionally, the cell cycle is segregated into four phases: DNA replication occurs during S phase, and chromosome segregation occurs during M phase. The S and M phases are separated by the so-called gap phases, G1 (before DNA replication) and G2 (before mitosis). We demonstrated that CCL21/CCR7 significantly altered cell cycle distribution such that more cells populated the G_2_/M phase. No significant differences were observed regarding the distribution of cells in the G_0_/G_1_ and S phases.

The cell cycle is regulated by cyclins and CDKs. D-type cyclins are regarded as key regulators of G_1_ progression [46]. Cyclin E is required for the G_1_/S transition [47], [48] and cyclin A is essential for progression through the S phase [49], [50]. Both cyclin A and the B-type cyclins associate with CDK1 to promote entry into mitosis [51], [52]. In this study, both protein and mRNA levels of cyclin A, cyclin B1, and CDK1 were significantly upregulated when cells were treated with CCL21 for 24 h. This indicates that CCL21/CCR7 accelerates the G_2_/M phase progression to promote cell proliferation. No significant differences in cyclin D1 or cyclin E expression levels were measured, indicating that CCL21/CCR7 has no significant effect on the G_0_/G_1_ phase or the G_1_/S transition. This finding demonstrates for the first time that CCL21/CCR7 has an impellent effect on cell cycle progression involving the G_2_/M phase.

Several studies using other cell types have indicated that activation of CCR7 is associated with enhanced cell survival via increased phosphorylation of ERK, JNK, or Akt [16]–[20], [33], [35]–[42]. We examined whether CCL21/CCR7 may enhance the expression of MAPK components and Akt in A549 and H460 cells. Our results suggested that CCL21/CCR7 enhanced the phosphorylation of ERK but not JNK, p38, or Akt. These findings are inconsistent with previously published reports that suggested a facilitative effect of CCR7 on the Akt, but not ERK, pathway [16], [19]. A separate study indicated that Akt is possibly upstream of ERK [43]. We found that CCL21/CCR7 still could significantly upregulate the expression of P-ERK after the cells were treated with LY294002 for 1 h. Because LY294002 selectively inhibits PI3K to prevent downstream activation of Akt, our results strongly suggest that Akt does not play a critical role in the interaction between CCL21/CCR7 and ERK. This is in concordance with a previous report [53]. The reason for these discrepancies remains unclear.

Since CCL21/CCR7 was capable of increasing the expression of P-ERK, we sought to determine whether there is an interaction between P-ERK and cyclin A, cyclin B1, or CDK1. Coimmunoprecipitation and reciprocal immunoprecipitation results strongly suggested an interaction between P-ERK and cyclin A, cyclin B1, or CDK1, especially in the presence of CCL21. This interaction could be weakened by inhibiting ERK with PD98059. In addition, PD98059 abolished the effect of CCL21/CCR7 on A549 and H460 cell proliferation and G_2_/M phase progression, as well as downregulating the expression of P-ERK, cyclin A, cyclin B1, and CDK1. These results demonstrate that the effect of CCL21/CCR7 on cell proliferation and upregulation of cyclin A, cyclin B1, and CDK1 may occur via the ERK pathway in human NSCLC cells.

This study suggests that activation of CCR7 with CCL21 can significantly promote proliferation of NSCLC cells in a time-dependent manner involving cyclin A, cyclin B1, and CDK1, possibly via the ERK, but not the Akt, pathway. This information may help clarify the mechanisms of cancer cell survival and identify potential targets for treatment of NSCLC.

## Materials and Methods

### Cell culture and reagents

A549 and H460 cell lines from our previous published paper [32] were cultured in RPMI-1640 or DMEM-F12 supplemented with 10% HyClone fetal bovine serum (FBS) (ThermoFisher Scientific, Fremont, CA, USA) in an atmosphere of 5% CO_2_ at 37°C. Cells were grown in 75 cm^2^ culture flasks and harvested in a solution of trypsin-EDTA at the logarithmic growth phase.

Cyclin A, cyclin B1, cyclin D1, cyclin E, CDK1, P-ERK, ERK, P-JNK, JNK, P-p38, p38, P-Akt, Akt, IgG, and β-actin mouse or rabbit monoclonal antibodies were purchased from Santa Cruz Biotechnology (Santa Cruz, CA, USA). Recombinant human CCL21 was purchased from Pepro Tech (Rocky Hill, NJ, USA). siCCR7 and Lipofectamine 2000 were purchased from Invitrogen (Carlsbad, CA, USA). Cell Counting Kit-8 (CCK-8) was purchased from Dojindo Laboratories (Beijing, China). PD98059 and LY294002 were obtained from Sigma (St. Louis, MO, USA) and used at 50 µM or 100 µM (final concentrations), respectively, in accordance with previous reports [40], [54]. Protein A/G beads were obtained from Beyotime (Haimen, China).

### siRNA treatment of cells

A549 and H460 cells were plated onto 6 cm^2^ cell culture dishes and grown to 30–50% confluence before transfection with Lipofectamine 2000 as previously described [55]. The transfection efficiency was assessed by flow cytometry. Efficiencies of siCCR7 and non-silencing control siRNA were tested using Western blot and RT-PCR. The sequences of siCCR7 and control siRNA were: CCR7, 5′-GCGUCAACCCUUUCUUGUATT-3′ and 3′-UACAAGAAAGGGUUGACGCAG-5′; control, 5′-UUCUCCGAACGUGUCACGUTT-3′ and 3′-ACGUGACACGUUCGGAGAATT-5′.

### Cell proliferation assay

Cell proliferative activities were examined using CCK-8. A549 and H460 cells were seeded onto 96-well plates (1000 cells/well) and treated with CCL21 for 0, 24, 48, or 72 h. After treatment, CCK-8 was added to each well according to the manufacturer's instructions and incubated for 4 h at 37°C. The optical density (OD) value of each well was measured using a microplate reader (Spectra Thermo, Männedorf, Switzerland) with a test wavelength of 450 nm.

### Cell cycle analysis

After treatment with CCL21 for 24 h, cells were harvested and washed twice with cold phosphate-buffered saline (PBS) and fixed in 75% ethanol for 2 h at 4°C. The fixed cells were washed twice with 500 µL of cold PBS. Cells then were stained with 500 µL of propidium iodide (PI) staining solution (50 µg/mL PI, 0.1% Triton X-100, 200 µg/mL DNase-free RNase in PBS) for 30 min at room temperature in the dark. Ten thousand events per sample were acquired using a FACS-scan flow cytometer (Becton-Dickinson, San Jose, CA, USA), and the percentage of cells in G_0_/G_1_, S, and G_2_/M phases of the cell cycle were determined using Modfit LT 3.0 (Becton-Dickinson).

### Western blot analysis

After treatment with CCL21 for 24 h, cells were extracted with lysis buffer (150 mM NaCl, 1% NP-40, 0.1% SDS, 2 µg/mL aprotinin and 1 mM PMSF) for 30 min at 4°C. Extracts were centrifuged at 12,000× *g* for 15 min at 4°C. Supernatants containing total protein then were harvested. Aliquots, each containing 50 µg protein, were separated by 10% SDS-PAGE and transferred to PVDF membranes at 55 V (cyclin A, cyclin B1, P-Akt, Akt) or 40 V (the others) for 2.5 h at low temperature. The membranes were blocked in 5% skim milk for 2 h, and proteins were detected using monoclonal antibodies at 1∶1000 (P-JNK, JNK, P-p38, p38 and β-actin) or 1∶200 (the others) dilution overnight at 4°C. Proteins were visualized using anti-mouse or anti-rabbit IgG conjugated with horse radish peroxidase (HRP) at 1∶6000 or 1∶8000 dilution for 2 h at room temperature, respectively. Bands were imaged with an EC3 Imaging System (UVP LLC, Upland, CA, USA), and the OD was measured using ImageJ (NIH, Bethesda, MD, USA). The OD difference between tested proteins and β-actin of the same sample was calculated as relative content and expressed graphically.

### RT-PCR and real-time PCR

Total RNA was isolated from cells using TRIzol (Invitrogen) according to the manufacturer's instructions. β-Actin was used as an internal control. To determine the efficiencies of siCCR7 and control siRNA, semi-quantitative RT-PCR was performed on a G-STORM thermal cycler (GRI Ltd, Byfleet, UK) using the TaKaRa RNA PCR Kit (AMV) Ver.3.0 (TaKaRa, Dalian, China). PCR primers were as follows: CCR7 F: 5′-GAGGCTATTGTCCCCTAAACC-3′, R: 5′-TGGAGGACAGTGAAGAAAACG-3′. The CCR7 amplicon length was 305 bp. β-actin F: 5′-AAATCGTGCGTGACATTAA-3′, R: 5′-CTCGTCATACTCCTGCTTG-3′. The β-actin amplicon length was 513 bp. The heat cycling conditions were as follows: 30 cycles of 40 s each, including denaturation at 95°C, annealing at 53°C, and extension at 72°C.

Real-time PCR was performed on an ABI Prism 7900HT Fast System (Applied Biosystems, Foster, CA, USA) using SYBR Premix Ex Taq II (TaKaRa). Amplifications were carried out in a total volume of 20 µL and cycled 40 times after initial denaturation (95°C for 30 s) with the following parameters: 95°C for 5 s and 60°C for 30 s. β-actin F: 5′-AGCACAGAGCCTCGCCTTTG-3′, R: 5′-ACATGCCGGAGCCGTTGT-3′. The β-actin amplicon length was 107 bp. Other primer sequences have been published in another study [56]. The reliability of PCR results was supported by analyzing the dissociation curve. Real-time PCR data were calculated using the 2^−ΔΔCT^ method on the SDS 2.4 software package (Applied Biosystems) [57].

### Coimmunoprecipitation

After treatment with CCL21 for 24 h, cells were extracted with lysis buffer (10 mM KCl, 1.5 mM MgCl_2_, 10 mM HEPES [pH 7.9], 1 mM PMSF, 1 mM DTT) and homogenized for 30 min at 4°C. The extracts were centrifuged at 12,000× *g* for 15 min at 4°C, and the supernatants containing total protein were harvested. Equal amounts of protein were exposed to antibodies against P-ERK, IgG, cyclin A, cyclin B1, or CDK1, which were immobilized on protein A/G beads. Following 3 h incubation at 4°C with gentle rotation, beads were washed extensively five times with lysis buffer, boiled, and microcentrifuged. Proteins were detected with antibodies against P-ERK, cyclin A, cyclin B1, or CDK1 by Western blot.

### Statistical analysis

Data were analyzed using SPSS 16.0 software. One-way analysis of variance (ANOVA) was used to evaluate the differences between groups with various treatments, and the least significant difference (LSD) test or Dunnett T3 test was used for post hoc subgroup analysis. Polynomial contrast was used for trend analysis. All data are presented as the mean ± SD of three independent experiments. Results were considered statistically significant for p<0.05. N-fold values for gene expression change up to 0.5 and below 2 were taken as nonsignificant in accordance with values obtained from negative control genes [57], [58].
